# Association of alleles carried at *TNFA *-850 and *BAT1 *-22 with Alzheimer's disease

**DOI:** 10.1186/1742-2094-5-36

**Published:** 2008-08-20

**Authors:** Anastazija Gnjec, Katarzyna J D'Costa, Simon M Laws, Ross Hedley, Kelvin Balakrishnan, Kevin Taddei, Georgia Martins, Athena Paton, Giuseppe Verdile, Samuel E Gandy, G Anthony Broe, William S Brooks, Hayley Bennett, Olivier Piguet, Patricia Price, Judith Miklossy, Joachim Hallmayer, Patrick L McGeer, Ralph N Martins

**Affiliations:** 1 Centre of Excellence for Alzheimer's Disease Research and Care, Faculty of Computing, Health and Science, School of Exercise, Biomedical and Health Sciences, Edith Cowan University, Joondalup Drive, Joondalup, 6027, WA, Australia; 2 Sir James McCusker Alzheimer's Disease Research Unit, School of Psychiatry and Clinical Neurosciences, University of Western Australia, Hollywood Private Hospital, Nedlands, 6009, WA, Australia; 3 Mount Sinai School of Medicine, New York, New York, 10029, USA; 4 Prince of Wales Medical Research Institute, UNSW, Barker Street, Randwick, NSW 2031, Australia; 5 Centre for Education and Research on Aging, University of Sydney and Concord Repatriation General Hospital, Concord, NSW, 2139, Australia; 6 School of Surgery and Pathology, University of Western Australia, Nedlands, Australia; 7 Department of Clinical Immunology and Biochemical Genetics, Royal Perth Hospital, Perth, WA, 6000, Australia; 8 Kinsmen Laboratory of Neurological Research, Department of Psychiatry, University of British Columbia, 2255 Wesbrook Mall, Vancouver, BC, V6T 1Z3, Canada; 9 Department of Genetics, and Center for Narcolepsy, Department of Psychiatry, Stanford University, Stanford, CA, 94305, USA

## Abstract

**Background:**

Inflammatory changes are a prominent feature of brains affected by Alzheimer's disease (AD). Activated glial cells release inflammatory cytokines which modulate the neurodegenerative process. These cytokines are encoded by genes representing several interleukins and *TNFA*, which are associated with AD. The gene coding for HLA-B associated transcript 1 (*BAT1*) lies adjacent to *TNFA *in the central major histocompatibility complex (MHC). BAT1, a member of the DEAD-box family of RNA helicases, appears to regulate the production of inflammatory cytokines associated with AD pathology. In the current study *TNFA *and BAT1 promoter polymorphisms were analysed in AD and control cases and BAT1 mRNA levels were investigated in brain tissue from AD and control cases.

**Methods:**

Genotyping was performed for polymorphisms at positions -850 and -308 in the proximal promoter of *TNFA *and position -22 in the promoter of *BAT1*. These were investigated singly or in haplotypic association in a cohort of Australian AD patients with AD stratified on the basis of their *APOE *ε4 genotype. Semi-quantitative RT-PCR was also performed for BAT1 from RNA isolated from brain tissue from AD and control cases.

**Results:**

*APOE *ε4 was associated with an independent increase in risk for AD in individuals with *TNFA *-850*2, while carriage of *BAT1 *-22*2 reduced the risk for AD, independent of *APOE *ε4 genotype. Semi-quantitative mRNA analysis in human brain tissue showed elevated levels of *BAT1 *mRNA in frontal cortex of AD cases.

**Conclusion:**

These findings lend support to the application of *TNFA *and *BAT1 *polymorphisms in early diagnosis or risk assessment strategies for AD and suggest a potential role for BAT1 in the regulation of inflammatory reactions in AD pathology.

## Background

Inflammation is a prominent pathological feature of the Alzheimer's disease (AD) brain, and might be initiated by the extracellular accumulation of amyloid β (Aβ) peptide [1]. Activated microglia and astrocytes cluster around the Aβ deposits and neurofibrillary tangles of AD brains and can release neurotoxic agents, including complement proteins and pro-inflammatory cytokines, such as interleukin (IL)-1β, IL-6 and tumor necrosis factor-alpha (TNFα) [2]. Polymorphisms in genes encoding IL-1α, IL-1β, IL-6 and TNFα correlate with heightened risk of AD [3]. For example, *IL1B *-511 [4], *IL6 *-174 [5] and *TNFA *-308 [6,7] associate with increased or reduced risk of AD. We showed that the *IL1A *-889 T/T and *IL1B *+3954 T/T genotypes mark increased risk for late-onset Alzheimer's disease (LOAD) in an Australian cohort [8].

When investigating potential genetic risk factors for AD pathology it is important to include established genetic risk factors. The most widely accepted genetic risk factor for late onset-forms of AD (LOAD) is the ε4 allele of the gene encoding apolipoprotein E (*APOE *ε4) [9,10]. Two recent studies have explored a potential association between *APOE ε*4 and the *TNFA *-850T (*2) promoter polymorphism in Irish [11] and Spanish [12] cohorts with conflicting outcomes. While in the Irish cohort possession of the *TNFA *-850*2 allele significantly increased the risk of dementia associated with *APOE *ε4 [11], no such synergistic effect was detected in the Spanish cohort [12] suggesting that the effect could be population specific or that other genetic or environmental factors may also play a contributing role. The availability of *APOE *genotype data from previous studies conducted by our research group [13,14] enabled us to investigate the potential link between *APOE *ε4 and *TNFA *-850*2 in a well characterised Australian cohort.

*TNFA *-308*2 (A allele) marks susceptibility to several autoimmune and inflammatory disorders (for a review see [15]) and has higher transcriptional activity than *TNFA *-308*1 (G allele) [16,17]. However *TNFA *-308*2 and linked alleles may mark increased risk [6,18] or protection [7,19] against AD, so we investigated *TNFA *-308 alleles singly or in haplotypic combination with polymorphisms in adjacent candidate genes to elucidate associations of these polymorphisms or haplotypic combinations of the respective alleles with AD pathology in an Australian cohort.

HLA-B associated transcript 1 (BAT1) is implicated in the regulation of several AD-associated cytokines [20,21]. BAT1 is a member of the DEAD-box family of RNA helicases, encoded in the central major histocompatibility complex (MHC) near to *TNFA *[22]. Members of this family are a group of highly conserved proteins involved in unwinding of RNA secondary structures [23]. DEAD-box proteins have been implicated in a number of different processes involving RNA such as mRNA stabilization [24]. Studies of anti-sense transfectants suggest BAT1 may act as a negative regulator of pro-inflammatory cytokines, namely IL-1, IL-6 and TNFα [20]. Furthermore, *BAT1 *promoter polymorphisms located at positions -22 and -348 can influence transcription through differential binding of transcription factors [21]. The C allele at *BAT1 *-22 (*BAT1 *-22*2) is found on a conserved ancestral haplotype associated with an increased risk of immunopathology (HLA-A1, B8, *TNFA *-308*2, DR3, DQ2) [21]. Neither *TNFA *-308*2 nor *BAT1 *-22*2 are unique to this haplotype, but when carried together form a haplospecific marker of a conserved block of the central MHC [25]. Here we present data from an investigation of associations between AD, the *APOE *ε4 genotype and carriage of *TNFA *-308*2, *TNFA *-850*2 and *BAT1 *-22*2 in a well-characterized Australian cohort. In addition, we report on *BAT1 *mRNA levels examined in frontal cortex (Fc) brain tissue from AD and control cases in order to investigate whether changes in *BAT1 *expression are associated with AD.

## Methods

### Genotyping

Alleles carried at *BAT1 *-22 (G→C) and *TNFA *-308 (G→A) and *TNFA *-850 (C→T) promoter polymorphisms was determined in 631 individuals from a population of Northern European descent (97% Caucasian). There were 359 control donors (45.7% females) with age at venipuncture of 76.7 ± 13.1 years (mean ± SD) and 272 AD cases (59.2% females, age: 77.1 ± 10.5). 391 cases were patients recruited from a memory clinic in Perth, Western Australia (226 AD cases and 165 controls). The remainder of patients were participants in the Sydney Older Persons Study; a random sample of community-dwelling people aged 75 and over at recruitment. Of these, 46 were classified as having AD at assessment, while 194 had no cognitive impairment and were used as controls for this analysis. All studies were conducted with approval from the institutional ethics committees and with informed consent of the participants. Methods of recruitment, diagnostic criteria and *APOE *genotyping were as described [13,14,26,27].

**Figure 1:**
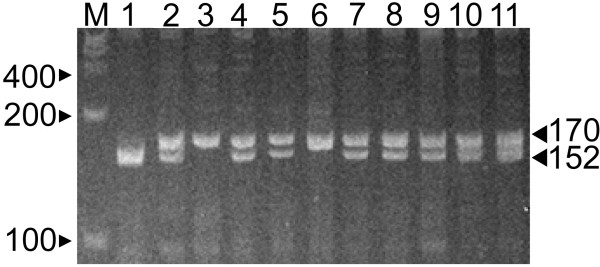
***BAT1 *-22 G/C promoter polymorphism genotyping**. A representation of a typical -22 C/G genotyping gel produced after digested PCR product was run on an 8% non-denaturing PAGE gel. M = Marker (100 base pair marker – arrows represent 400, 300 and 200 bp fragments). Black arrowheads correspond to allele fragments: -22 C = 152 bp & 18 bp, and -22 G = 170 bp. Lane 1 = -22 CC genotype. Lanes 2,4,5,7,8,9,10 and 11 = -22 CG genotype. Lanes 3 and 6 = -22 GG genotype.

Genomic DNA was extracted from peripheral lymphocytes using a standard protocol [28]. *BAT1 *-22 alleles were determined by PCR amplification in a total volume of 20 μL, containing 1.0 U of *Taq *polymerase (Fisher Biotec, Australia), 0.2 mM each dNTP and 3.0 mM MgCl_2_, on a Mastercycler Gradient thermal cycler (Eppendorf, Germany) as follows: 1 cycle of 95°C for 5 minutes, 44 cycles of 95°C for 30 seconds, 56°C for 35 seconds and 72°C for 40 seconds, followed by 1 cycle of 72°C for 10 minutes. The oligonucleotide primers, (P1) 5'-CAACCGGAAGTGAGTGCA -3' and (P2) 5'-CAGACCATCGCCTGTGAA-3', were purchased from Genset Pacific Pty. Ltd (Lismore, Australia). Amplicons were digested at 37°C using 5 U *Alw*44I (restriction sequence GTGCAC), separated on 8% non-denaturing polyacrylamide gel at 110 V for 1.5 hours and stained with ethidium bromide to reveal DNA fragments with migration patterns specific for each allele (Allele 1 (G) = 170 base pairs (bp); Allele 2 (C) = 152 bp and 18 bp; Figure 1).

*TNFA *-308 alleles were determined via PCR amplification in a total volume of 20 μL, containing 0.6 U *TAQti *(Fisher Biotec, Australia), 0.2 mM each dNTP, 1.5 mM MgCl_2 _and 0.5 mg/ml BSA amplified as follows: 1 cycle of 94°C for 2 minutes, 35 cycles of 94°C for 30 seconds, 63°C for 30 seconds and 72°C for 30 seconds, followed by 1 cycle of 72°C for 5 minutes. Primers, (P1) 5'-AGGCAATAGGTTTTGAGGGCCAT-3' (underline denotes mismatch) and (P2) 5'-TCCTCCCTGCTCCGATTCCG-3', were purchased from Proligo Pty. Ltd (Lismore, Australia). Amplicons were digested at 37°C using 3 U *NcoI *(restriction sequence C▲CATGG), separated on 5% high resolution agarose gels at 280 V (12 minutes) and stained with ethidium bromide to reveal fragments with migration patterns specific for each allele (Allele 1 (G) = 88 bp and 19 bp; Allele 2 (A) = 107 bp).

*TNFA *-850 alleles were determined via PCR amplification in a total volume of 20 μL, containing 0.6 U of *TAQti *polymerase (Fisher Biotec, Australia), 0.2 mM each dNTP, 1.5 mM MgCl_2 _and 0.5 mg/ml BSA as follows: 1 cycle of 94°C for 3 minutes, 35 cycles of 94°C for 45 seconds, 60°C for 30 seconds and 72°C for 45 seconds, followed by 1 cycle of 72°C for 5 minutes. Primers were modified from those initially published [27]. (P1) 5'-TCGAGTATCGGGGACCCCCCGTT-3' (underline denotes mismatch) and (P2) 5'-CCAGTGTGTGGCCATATCTTCTT-3' were purchased from Proligo Pty. Ltd (Lismore, Australia). Amplicons were digested at 37°C using 3 U *HincII *(restriction sequence GTT▲AAC), separated on a 5% high resolution agarose gels at 280 V (12 minutes) and stained with ethidium bromide to reveal DNA fragments with migration patterns specific for each allele (Allele 1 (C) = 105 bp and 23 bp; Allele 2 (T) = 128 bp) [29].

### Brain tissue samples

Total RNA and protein was isolated from brain tissue (frontal cortex) samples from subjects with histopathologically confirmed definite AD and control cases without any AD pathology. Autopsy was performed within 48 hours after death. Subjects with PS1 mutations and a number of familial AD cases with *APOE *ε4 genotypes were from local pedigrees and from the brain tissue bank of Drexel University College of Medicine (Philadelphia, PA, USA). Control brain tissue was obtained locally (Western Australia) and tissues were also received from the New South Wales (NSW) Tissue Resource Centre (Sydney, NSW, Australia), which is supported by The University of Sydney, Neuroscience Institute of Schizophrenia and Allied Disorders, National Institute of Alcohol Abuse and Alcoholism and NSW Department of Health.

### RNA extraction and semi-quantitative RT-PCR

Total RNA was isolated using Trizol^® ^(Gibco BRL, Grand Island, New York, USA) according to manufacturer's instructions. RNA was extracted from 100 mg of frontal cortex brain tissue from 12 cases with familial AD either with PS1 mutations or linked to inheritance of the APOE-ε4 allele (mean age at time of death: 63 years, range: 50 – 77) and from 16 control cases without AD pathology (mean age at time of death: 50.25 years, range: 18 – 74 years). RNA concentrations were determined spectrophotometrically and 1 μg aliquots were reverse transcribed using the Omniscript™ Reverse Transcriptase Kit (QIAGEN; Victoria, Australia).

Primers required to assess the expression of *BAT1 *and *β-ACTIN *mRNA were purchased from Genset Pacific Pty. Ltd (Lismore, Australia): BAT1(F): 5'-AGAGGCTCTCTCGGTATCA-3', BAT1(R): 5'-GCTGATGTTGACCTCGAAA-3', BACTIN(F): 5'-TGGAATCCTGTGGCATCCATGAAAC-3', BACTIN(R): 5'-TAAAACGCAGCTCAGTAACAGTCCG-3'. Primers for glyceraldehyde-3-phosphate dehydrogenase (*GAPDH*) were as previously described [30]. 5 μL cDNA was amplified in a 20 μL reaction on a LightCycler™ (Roche, USA). Each 20 μL PCR reaction contained 1.25 mM dNTP, 20 pmol each primer, 0.25 mg/mL BSA, 1.5 units *Taq *Platinum polymerase and 0.5 × SYBR Green (Invitrogen, USA). Amplifications of cDNA were performed as follows: Denaturation at 95°C for 5 minutes, followed by amplification with 44 cycles at 94°C for 30 seconds, annealing (62°C for *BAT1*, 64°C for *β-ACTIN*, and 65°C for *GAPDH*) for 15 seconds and 72°C for 40 seconds. Amplicons were separated on 1% TBE agarose gels and visualised by ethidium bromide staining. The quantification of cDNA was achieved with SYBR Green I dye (Sigma, USA).

Standard curves were generated using 10-fold dilutions of a previously purified bulk cDNA PCR product (stored at a concentration of 1 ng/μL) and analysed using a 'fit points' method with the LightCycler™ run software, version 4.0. Melting curve analyses were used to confirm the generation of a single product. This was further confirmed by agarose gel electrophoresis. The amplified *BAT1 *PCR products were sequenced using big-dye terminator chemistry on an ABI automated DNA sequencer (ABI, USA) to confirm the specific amplification of *BAT1*. The house keeping genes *β-ACTIN *and *GAPDH *were used for normalization of *BAT1 *mRNA expression. Statistical significance analysis was performed using the Mann-Whitney U test.

The Statistical Package for Social Sciences (SPSS version 11.5; SPSS Inc., Chicago, Illinois, USA) was used to establish genotype and allele frequencies and to check for Hardy-Weinberg equilibrium (HWE). Initial data comparison involved Pearson's χ^2 ^and odds ratio (OR) analysis of two by two contingency tables to compare the relative genotype frequencies in AD and control groups. SPSS was further employed to perform Cochran Armitage testing for trends where assumptions of HWE were not met. The same programme was also used to perform direct logistic regression analysis, where all variables were entered into the equation simultaneously to determine the overall contribution of each genotype on AD in this cohort, whilst controlling for established AD risk factors (age and gender). Estimation of linkage disequilibrium and analysis of haplotypes was performed using Thesias [31].

GenBank codes for genes investigated in this study include *APOE *(MIM: 107741, GeneID: 348), *TNFA *(MIM: 191160, GeneID: 7124) and *BAT1 *(MIM: 142560, GeneID: 7919).

## Results

Pearson's chi-square (χ^2^) and Odds ratio (OR) analysis of the *BAT1 *-22 1/1 and 1/2 genotypes revealed a significant association between a complete absence of the *BAT1 *-22*2 allele and AD (Table 1). However, this apparent level of protection afforded by the *BAT1 *-22*2 allele revealed no gene dosage effect and was limited to homozygosity of this allele (Table 1). Pearson's χ^2 ^and OR analysis of the *TNFA *-308 single nucleotide polymorphism (SNP) revealed a weak yet mildly significant trend whereby possession of the -308*2 allele conferred protection from the development of AD. However, this was only significant when allele frequencies were analysed (Table 1). No significant protective effect was observed when genotype frequencies were analysed. Pearson's χ^2 ^and OR analysis of genotype and allele frequencies from data generated through the genotyping of the *TNFA *-850 SNP revealed a strong association of the *TNFA *-850*2/2 genotype and the *TNFA *-850*2 allele with an increased risk for AD (Table 1).

**Table 1:** Analysis of Genotype and Allele frequencies of the *BAT1 *-22, *TNFA *-308 and *TNFA *-850 polymorphisms

Marker	Genotype or allele	Ctrl numbers (%)	AD numbers (%)
*BAT1 *-22	1/1	144 *(40.1)*	117 *(43.0)*
	1/2	167 *(46.5)*	138 *(50.7)*
	2/2	48 *(13.4)*	17 *(6.3)*^a^
	1	455 *(63.4)*	372 *(68.4)*
	2	263 *(36.6)*	172 *(31.6)*
*TNFA *-308	1/1	226 *(63.0)*	188 *(69.1)*
	1/2	104 *(29.0)*	70 *(25.7)*
	2/2	29 *(8.0)*	14 *(5.1)*
	1	556 *(77.4)*	446 *(82.0)*
	2	162 *(22.6)*	98 *(18.0)*^b^
*TNFA *-850	1/1	287 (79.9)	183 (67.3)
	1/2	61 (17.0)	70 (25.7)
	2/2	11 (3.1)	19 (7.0)^c^
	1	635 (88.4)	436 (80.1)
	2	83 (11.6)	108 (19.9)^d^

Ctrl = Control cases without AD pathology

AD = Alzheimer's disease cases

^a ^*BAT1 *-22*2/2 *versus *non-2/2 in AD, *P *< .005 (Pearson χ^2 ^= 8.49) OR = 0.43 (95% CI = 0.24 – 0.77).

^b ^*TNFA *-308*2 allele in AD, *P *= .048 (Pearson χ^2 ^= 3.91) OR = 0.75 (95% CI = 0.57 – 1.00).

^c ^*TNFA *-850*(2/2, 1/2) *versus *1/1 in AD, *P *< .001 (Pearson χ^2 ^= 13.06) OR = 1.94 (95% CI = 1.35 – 2.78.0).

^d ^*TNFA *-850*2 allele in AD, *P *< .001 (Pearson χ^2 ^= 16.57) OR = 1.90 (95% CI = 1.39 – 2.59).

By convention Pearson's χ^2 ^and OR analysis are commonly used to evaluate data generated from large genotyping studies and explore frequency distributions. However, in order for such analysis to produce meaningful outcomes strict conditions of HWE must be met. In the current study the distributions of *APOE *and *BAT1 *-22 alleles were in HWE (χ^2^, *P *= .54 and p = .97, respectively) within the control populations. However significant deviation from HWE within the control group populations was observed for *TNFA *-850 and *TNFA *-308 (χ^2 ^test, *P *< .005). Therefore, subsequent analyses employed Armitage's trend test (rather than Pearsons's χ^2 ^analysis), to correct for potential type I errors associated with departure from HWE [32].

Armitage's testing for trends revealed a significant association between *APOE *ε4 and AD (χ^2 ^= 108.91, *P *< 0.0001). *TNFA *-850*2 was also significantly associated with increased risk for AD while a significant protective trend was observed for *BAT1 *-22*2 (Table 2). The protective effect initially observed for *TNFA *-308*2 in the genotype and allele frequency distribution analysis (Table 1) did not reach significance using Armitage's test for trend (Table 2). This may reflect a haplotypic association with *BAT1 *-22*2 since the alleles are in linkage disequilibrium (LD) in the West Australian population [25].

**Table 2:** Armitage test for trend for *BAT1 *and *TNFA *genotypes

Marker	Genotype trend	χ^2^-value	*P*-value
*BAT1 *-22	1/1 < 1/2 < 2/2	7.26	<.05
*TNFA *-308	1/1 < 1/2 < 2/2	5.28	.07
*TNFA *-850	1/1 < 1/2 < 2/2	20.17	<.00005

Logistic regression analysis including age and gender associated *BAT1 *-22*2/2 with protection against AD, while *TNFA *-850*1/2 and *TNFA *-850*2/2 conferred risk (Table 3). These findings support Armitage's test for trend results and suggest a possible gene dosage effect for the presence of the TNFA -850*2 allele.

**Table 3:** Direct logistic regression analysis

Variable	Odds ratio	*P*-value	95.0% C.I.
*BAT1 *-22*2/2^a^	0.436	<.01	0.238 – 0.798
*TNFA *-850*1/2^b^	1.8	<.005	1.218 – 2.669
*TNFA *-850*2/2^c^	2.709	<.05	1.260 – 5.824

Direct logistic regression model with Odds ratios representing risk assessment for AD.

^a ^Homozygosity of *BAT1 *-22*2 allele (with absence of allele as reference).

^b ^Heterozygosity of *TNFA *-850*2 allele (with absence of allele as reference).

^c ^Homozygosity of *TNFA *-850*2 allele (with absence of allele as reference).

Additional logistic regressions analysis of interaction terms between *APOE *ε4 and the *TNFA *and *BAT1 *SNPs showed no interactions between the effects marked by *APOE *ε4, and *BAT1 *-22*2/2, *TNFA *-850*1/2 or *TNFA *-850*2/2. Furthermore, a stratified analysis based on *APOE *genotype using the Mantel-Haenszel technique showed no significant differences in Odds ratios when estimating effects on AD risk of individual SNPs *versus *a combination of these SNPs with *APOE *ε4. This suggests that the observed protective effect of *BAT1 *– 22*2/2 and the increased risk associated with *TNFA *-850*2 are independent of *APOE *ε4 genotype.

*BAT1 *and *TNFA *are located in close proximity within the MHC [21,22] and their alleles are in marked LD [25]. Therefore, the computer programme Thesias [31] was used to generate LD matrices for analysis of LD and for haplotype analysis. *BAT1 *-22, *TNFA *-308 and *TNFA *-850 were all in LD, so haplotype frequencies were estimated under LD for all three markers and combinations of two markers. The only significant result was obtained for *BAT1 *-22*1 in combination with *TNFA *-850*2 (OR = 1.54, *P *< 0.05). However, the individual Odds ratios for *TNFA *-850*1/2 and *TNFA *-850*2/2 were higher than for the above haplotype (i.e. individual OR for *TNFA *-850*1/2 = 1.8 and for *TNFA *-850*2/2 = 2.7). This indicates that the presence of *BAT1 *-22*1 in haplotypic association with *TNFA *-850*2 cannot explain the risk effects conferred by *TNFA *-850*2. Therefore, both the protective effect associated with *BAT1 *-22*2 and the increased risk associated with *TNFA *-850*2 are more likely due to the individual SNPs themselves or a potential haplotypic association with other genes.

**Figure 2:**
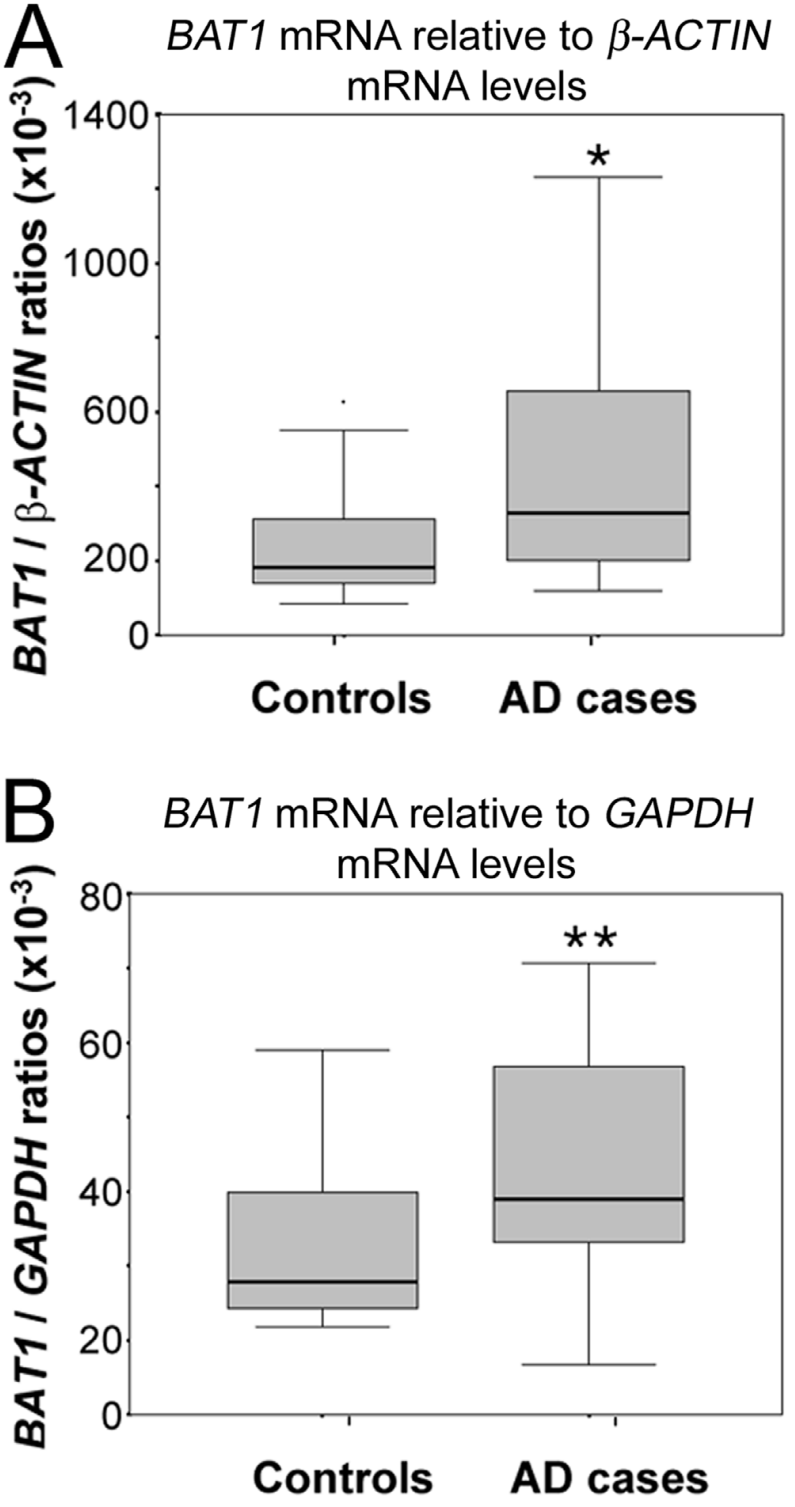
**Semi-quantitative RT-PCR of *BAT1 *and *DDXL *mRNA in frontal cortex of AD (n = 12) and control cases (n = 16)**. Data is represented as Box-plots showing median values and quartiles. (A) *BAT1 *mRNA levels normalized against *β-ACTIN *(Mann-Whitney U test: *P = .037), (B) *BAT1 *mRNA levels normalized against *GAPDH *(Mann-Whitney U test: **P = .057).

In order to test whether transcription of *BAT1 *and the homologous gene *DDXL *was altered in AD, mRNA levels of both BAT1 and DDXL were examined in brain frontal cortex tissue of AD and control cases. Analysis of *BAT1 *mRNA levels (Figure 2) revealed significantly elevated mRNA levels for *BAT1 *normalized against *β-ACTIN *(a) while normalization with *GAPDH *(b) showed marginal significance for increased *BAT1 *mRNA levels in the AD brains (Mann-Whitney U test: *P *= .037 and *P *= .057 respectively).

## Discussion

AD is a multifactorial disorder with a number of alterations in the immune profile occurring during disease progression in both the brain [33] and the periphery [34,35]. Recently studies have reported links between risk for AD and polymorphisms in the promoter regions of *TNFA *at positions -308 [6,18] and -850 [11]. The current study utilized a well characterised sample to investigate these potential associations in an Australian cohort. In addition, BAT1 has been implicated in modulation of inflammatory cytokines [20]. Therefore, the current study investigated alleles of the BAT1 -22 promoter polymorphism as a potential risk factor for AD, singly or in haplotypic association with the *TNFA *promoter polymorphisms.

Analysis of individual SNPs revealed no significant association between AD and *TNFA *-308*2. This contrasts with reports in the literature that associate the *TNFA *-308*2 allele with either increased risk for AD [6,18] or protection against this disorder [7,19]. While data from the current study appears to be more supportive of a potential protective role for *TNFA *-308*2 against AD (Table 1), no conclusions can be drawn solely based on genotype and allele frequency analysis due to control group deviations from HWE that might affect the rate of type I error. However, it is possible that the inconclusive result obtained for *TNFA *-308*2 may be due to haplotypic associations of this polymorphism with other MHC markers such as the BAT1-22*2 allele.

In contrast to the ambiguous result obtained for *TNFA *-308*2, analysis of individual SNPs revealed that *TNFA *-850*2 was clearly significantly associated with increased risk for AD. The literature shows association of the TNFA -850*2 with vascular dementia [11] and individuals at high risk for dementia, such as those with Down's Syndrome [36]. However, a clear association of *TNFA *-850*2 with AD has only previously been reported as a synergistic effect in combination with *APOE *ε4 in a Northern Irish population [11], while a similar study in a population from Northern Spain failed to produce evidence in support of a synergistic effect between *TNFA *-850*2 and *APOE *ε4 [12]. The authors suggested that this discrepancy might reflect true genetic differences between the populations and pointed out that differences in allele frequency distributions between the two different European populations might indicate linkage disequilibrium between the *TNFA *-850 and another marker that might represent the true disease causing gene [12].

The current study presents data in support of the notion that *TNFA *-850*2 contributes to the risk of AD independently of the *APOE *ε4 allele. Furthermore, logistic regression analysis revealed a possible gene dosage effect with increase in copy numbers of the *TNFA *-850*2 allele leading to higher Odds ratios. It is, however, possible that a gene linkage with *TNFA *-850*2 would show a parallel OR pattern, and might account for the apparent gene dosage effect attributed to the *TNFA *-850*2 allele. Since all three markers investigated exerted their effects independently of *APOE ε*4 but were found to be in LD with one another, haplotype frequencies, taking into account LD between markers, were estimated for all three MHC markers and also for combinations of two markers in order to investigate whether an AD risk or protection associated haplotype could be responsible for the effects observed.

Only one haplotype (*BAT1 *-22*1 in combination with *TNFA *-850*2) appeared to be significantly associated with risk for AD, but the observed Odds ratio was lower for this haplotype (OR = 1.54) than the OR for the single polymorphisms associated with AD risk (*TNFA *-850*1/2, OR = 1.8 and *TNFA *-850*2/2, OR = 2.7). This indicates that, although in LD with the other two markers *TNFA *-850*2 did not exert its risk for AD through a haplotypic association with these polymorphisms. While it cannot be entirely ruled out that linkage disequilibrium with other as yet not identified markers may be responsible for the effect observed in this investigation, the current study identifies the *TNFA *-850*2 allele as a candidate marker that may confer risk for AD in the Australian population. Further investigation with larger participant numbers and in other populations is clearly warranted.

While the polymorphisms in the promoter regions of *TNFA *are likely to directly affect transcription of the *TNFA *gene, ultimate levels of TNFα protein in tissues can also be influenced by other regulating factors such as *BAT1*. In the current study BAT1-22*2/2 was significantly associated with protection against the development of AD. Similar to the association between increased risk for AD and the presence of the *TNFA *-850*2 allele, the protective effect of *BAT1*-22*2/2 was found to be independent of *APOE *ε4 status. Furthermore, none of the estimated haplotypic associations with the two *TNFA *markers that are in linkage disequilibrium with *BAT1 *have provided evidence to suggest that the effect observed for *BAT1*-22*2/2 is due to a haplotypic association with these markers. While the possibility remains that the protective BAT1 effect might be due to LD with another gene as yet not investigated, it is also possible that BAT1 might assert an independent effect on AD risk.

A potential independent role for BAT1 in AD pathology is supported by the notion that the *BAT1 *-22 polymorphism may not only have the potential to affect transcription of *BAT1 *but, through the role BAT1 plays in mRNA stabilization, this protein may also affect translation of a number of inflammatory cytokines linked to AD pathology, including *TNFA*. It has previously been reported that BAT1 plays a potential role in the regulation of inflammatory cytokines, including *TNFA *[20,21] and the *BAT1 *-22 allele has been associated with certain autoimmune disease susceptible ancestral haplotypes such as the 8.1 MHC AH amongst others [21]. Since BAT1 appears to regulate a number of inflammatory cytokines for which alterations are observed in AD pathology the current study is the first to provide evidence to show that a *BAT1 *promoter polymorphism is significantly associated with AD pathology.

It is of interest to note that for the *TNFA *-850 polymorphism the less frequent allele conferred risk for AD while the opposite was found for the less frequent allele (C) of the *BAT1 *-22 polymorphism which was associated with a decreased risk for AD. This finding that the *BAT1 *-22*2 (C) allele is associated with protection against AD is in contrast to the findings for autoimmune disorders where the less common number 2 allele is implicated with ancestral haplotypes that confer increased risk [20,21]. In order to explain this phenomenon it is important to gain a better understanding of the function of BAT1. The yeast homolog of BAT1, Sub2p, has been shown to be required for mRNA export through nuclear pores [37,38]. Previous findings have shown that the -22 C *BAT1 *allele, associated with the autoimmune disease susceptible 8.1 MHC ancestral haplotype, may result in reduced *BAT1 *transcription [21]. However, it has also been demonstrated that both injection of excess UAP56 (BAT1) into *Xenopus *oocytes as well as depletion of HEL, the *Drosophila *homologue of UAP56, by RNAi resulted in defects in mRNA export from the nucleus [39,40]. This indicates that both excess levels of BAT1 and a lack of this protein can lead to abnormalities in mRNA export and splicing. Hence, the presence of different alleles of *BAT1 *-22 may potentially lead to a range of different aberrations in mRNA processing resulting in a variety of different phenotypic manifestations of pathology. It is, therefore, possible that the *BAT *-22*2 allele *per se *may be protective against AD but still also be part of an array of SNPs that may confer risk for certain autoimmune disorders. The complexity of potential phenotypical effects as well as possible haplotypic associations of *BAT1 *-22 with other genes indicate that further studies are warranted to explore whether the *BAT1*-22*1 allele may confer an independent risk for AD other than just in haplotypic combination with *TNFA *-850*2 as observed in the current study.

Therefore, while the possibility of LD with other genes cannot be ruled out the current study provides evidence in support for a potential role for BAT1 in AD pathology. BAT1 -22 and TNFA -850 in combination with other biochemical and cognitive markers might serve as genetic markers for diagnostic purposes or AD risk assessment strategies. Moreover, in light of current international drug development research in the AD field, establishment of genetic profiles may help to identify individuals more likely to experience benefits from certain treatments or may prevent individuals genetically unfavourably predisposed from receiving costly, yet ineffective treatment. Since the SNPs investigated could also lead to functional differences it is of great importance to investigate phenotypical characteristics conferred by these polymorphisms.

Considering that *BAT1 *has a potential regulatory role for inflammatory cytokines [20,21] analysis of *BAT1 *mRNA and protein levels in AD brain tissue may reveal a functional role for the BAT1 protein in AD pathology. To investigate whether transcription of *BAT1 *was affected in AD, levels of *BAT1 *mRNA were determined in brain tissue from confirmed AD and control cases. This revealed significantly elevated levels of *BAT1 *and *DDXL *mRNA in Fc of AD cases and suggests a potential functional role for BAT1 in AD pathogenesis. It is not implausible to suggest that levels of BAT1 may rise as a response mechanism to counteract the inflammatory reactions that occur in regions of AD pathology. However, a repetition of this study with a larger sample size to enable parametric analysis of results may help to confirm the significance of these findings.

These data are of particular interest in light of recent findings that oligonucleotides spanning the promoter polymorphism -22 to -348 region of *BAT1 *autoimmune disease resistant 7.1 AH bind DNA/protein complexes as shown by electrophoretic mobility shift assays [41]. At position -22 these complexes appear to include the octamer binding protein family member, transcription factor Oct1 [39]. Oct1 has been shown to bind *TNFA *at position -857T and can interact with the pro-inflammatory NF-κB transcription factor p65 subunit [42]. As TNFα has been implicated in inflammation observed in AD brains [2] the above studies together with the current findings suggest an important association between *BAT1 *expression and regulation of inflammatory cytokines in the AD brain. The exact mechanisms of this link between *BAT1 *-22 promoter polymorphism and inflammatory reactions in the AD brain remain to be explored in future studies.

To establish the role of BAT1 in AD pathology it is imperative to examine levels of BAT1 in AD affected tissues in a larger number of cases. Apart from its presence in brain tissue, *BAT1 *mRNA transcripts have been detected in pancreas, kidney, skeletal muscle, liver, lung and heart [43]. The presence of BAT1 in hematopoietic cells [20] makes this protein a potential biomarker in early diagnosis or monitoring of progression of disorders with inflammatory responses, such as AD.

## Conclusion

The current study has revealed an *APOE *ε4 independent association of *TNFA *-850*2 with increased risk for AD, and an *APOE *ε4 independent association of *BAT1 *-22*2/2 with decreased risk for AD. These findings were not enhanced by haplotype analysis of polymorphisms in linkage disequilibrium suggesting that the observed effects may have resulted from the single SNPs. Hence, these SNPs may represent valuable markers in risk assessment, prognosis and therapeutic approaches for AD. In addition, the current study has provided evidence for a novel role for BAT1 in AD pathogenesis. BAT1 may play a role in regulating the inflammatory response in AD through influencing mRNA export and translation. Investigations of *BAT1 *promoter polymorphisms and mRNA and protein levels in other populations are clearly warranted to confirm this initial finding. Inflammatory processes form important underlying mechanisms in AD pathology. Elucidating the role of the currently investigated SNPs in AD pathology may contribute towards an understanding of the regulatory mechanisms of these events, and may provide new targets for drug development to combat AD.

## Competing interests

The authors declare that they have no competing interests.

## Authors' contributions

AG has isolated RNA from AD and control brain tissue and has been drafting and writing the manuscript, has performed data analysis for the mRNA work, and has been involved in interpretation of data and revising the manuscript critically for important intellectual content. KD has performed the semi-quantitative RT-PCR and data analysis and has made substantial contributions towards drafting the manuscript. SML has made substantial contributions towards genotyping, data analysis and interpretation and drafting of the manuscript. RH, KB and KT contributed towards the genotyping process. GM and AP have been involved in the sample acquisition and/or the DNA extraction process. GV and SEG have made substantial intellectual contributions towards the manuscript. GAB, WSB, HB and OP were involved in sample acquisition and processing. PP has made substantial contributions to the concept and design of the study and the manuscript as expert adviser, and has contributed towards data interpretation. JM contributed towards analysing brain tissue from a substantial proportion of the cases for histopathological diagnosis. JH has been critically involved in statistical analyses and interpretation of data, including genotype and haplotype analyses. PM has provided substantial expert advice with regard to analysis and interpretation of data and manuscript drafting. RNM has made the most substantial contributions towards the conception and design of the study and has given final approval of the version to be published. All of the authors have read and approved the final manuscript.
